# *Drosophila* lifespan control by dietary restriction independent of insulin-like signaling

**DOI:** 10.1111/j.1474-9726.2008.00373.x

**Published:** 2008-04-01

**Authors:** Kyung-Jin Min, Rochele Yamamoto, Susanne Buch, Michael Pankratz, Marc Tatar

**Affiliations:** 1 Department of Ecology and Evolutionary Biology, Division of Biology and Medicine, Brown University Providence, RI 02912, USA; 2 Institute for Genetics, Forschungszentrum Karlsruhe Karlsruhe, Germany

**Keywords:** aging, *Drosophila*, insulin/IGF-1 signaling, longevity regulation

## Abstract

Reduced insulin/insulin-like growth factor (IGF) signaling may be a natural way for the reduction of dietary nutrients to extend lifespan. While evidence challenging this hypothesis is accumulating with *Caenorhabditis elegans*, for *Drosophila melanogaster* it is still thought that insulin/IGF and the mechanisms of dietary restriction (DR) might as yet function through overlapping mechanisms. Here, we aim to understand this potential overlap. We found that over-expression of dFOXO in head fat body extends lifespan and reduces steady-state mRNA abundance of *insulin-like peptide-2* under conditions of high dietary yeast, but not when yeast is limiting. In contrast, conditions of DR that increase lifespan change only *insulin-like peptide-5* (*ilp5*) mRNA abundance. Thus, reduction of *ilp5* mRNA is associated with longevity extension by DR, while reduction of *insulin-like peptide-2* is associated with the diet-dependent effects of FOXO over-expression upon lifespan. To assess whether reduction of *ilp5* is required for DR to extend lifespan, we blocked its diet-dependent change with RNAi. Loss of the *ilp5* dietary response did not diminish the capacity of DR to extend lifespan. Finally, we assessed the capacity of DR to extend lifespan in the absence of dFOXO, the insulin/IGF-responsive transcription factor. As with the knockdown of *ilp5* diet responsiveness, DR was equally effective among genotypes with and without dFOXO. It is clear from many *Drosophila* studies that insulin/IGF mediates growth and metabolic responses to nutrition, but we now find no evidence that this endocrine system mediates the interaction between dietary yeast and longevity extension.

## Introduction

Dietary restriction (DR) extends the lifespan of many animals (Mobbs *et al*., 2007). Adult life expectancy of mice is increased up to 65% when calorie intake is reduced (Weindruch *et al*., 1986). Methods of DR with *Caenorhabditis elegans* include dilution of axenic liquid media, dilution of bacteria on agar plates and behavioral mutants that reduce feeding rate (Walker *et al*., 2005). These operations routinely increase lifespan by 30–80%. Similar lifespan extension occurs with *Drosophila melanogaster* by diluting dietary yeast in adult nutrient media, and to a lesser extent by diluting dietary sugar (Chippindale *et al*., 1993; Chapman & Partridge, 1996; Carvalho *et al*., 2005). From these and other examples, longevity extension by DR appears to be a nearly universal animal trait, and may therefore occur through conserved physiological and molecular mechanisms. Accordingly, it is widely thought that we may learn a great deal about the regulation and causes of aging through the analysis of DR in invertebrate model systems.

A powerful approach for this purpose is to measure the ability of restricted nutrients to extend lifespan in genotypes lacking specific molecular functions. When DR induces the same degree of longevity extension in a mutant and its corresponding wild-type, DR must work independent of the gene's product. Conversely, when longevity extension is reduced or eliminated by a mutation, the product of the tested gene may participate in the mechanism by which DR extends lifespan. Applications of this approach have been recently reviewed for *C. elegans* and *D. melanogaster* (Partridge *et al*., 2005; Walker *et al*., 2005; Houthoofd *et al*., 2007; Tatar, 2007). Notably, analyses have aimed to determine whether longevity extension by DR involves insulin/insulin-like growth factor (IGF) signaling (IIS) because mutations of this pathway extend lifespan, and this endocrine system affects the nutrient responses of metabolism, growth and reproduction.

In *C. elegans*, mutants at the locus *eat* have reduced pumping of the pharynx, and correspondingly they eat less and are long lived (Lakowski & Hekimi, 1998). This longevity extension appears to be insulin/IGF independent because lifespan is still extended when *eat* is combined with mutants of *daf-16*, the FOXO transcription factor that is inactivated by signal transduction through the insulin/IGF receptor encoded by *daf-2* (Lakowski & Hekimi, 1998). Likewise, longevity extension in diluted axenic media is effective in wild-type and null genotypes of *daf-16* (Houthoofd *et al*., 2003). Overall, as *daf-16* is required for *daf-2* mutation to extend lifespan under normal food conditions, these data suggest that the mechanisms of DR in *C. elegans* are somehow independent of IIS. Clues to as what might fulfill the signaling of diet restriction are emerging from analysis of forkhead transcription factors other than *daf-16* (Panowski *et al*., 2007).

These conclusions with *C. elegans* differ from the prevailing view for *Drosophila* (Clancy *et al*., 2002; Partridge *et al*., 2005). Yeast restriction in the fly robustly extends lifespan of both male and female flies, as do mutations of the insulin receptor (InR) and of the InR substrate homolog encoded by *chico* (Clancy *et al*., 2001; Tatar *et al*., 2001; Tu *et al*., 2002). Over-expression of dFOXO in adult fat body extends lifespan and simultaneously reduces mRNA of insulin-like peptide-2 (*ilp2*) but not of *ilp3* or *ilp5* produced in the medial secretory neurons (MSNs) of the adult brain (Hwangbo *et al*., 2004). Likewise, reduction of Jun kinase (JNK) signaling in the MSN extends lifespan and represses *ilp2* (Wang *et al*., 2005). Furthermore, ablation of the MSN is sufficient to increase survival, lipids and carbohydrates (Wessells *et al*., 2004; Broughton *et al*., 2005), suggesting that insulin-like peptides from the MSN are key regulators of both longevity and metabolism. Consistent with these responses, insulin-like peptides are secreted from the MSN to induce a cascade of FOXO-mediated transcriptional responses when sugar-fed adults are switched to sugar–yeast diet (Gershman *et al*., 2007). Finally, in the sole report to directly test for interaction of DR and IIS, longevity extension upon restricted media was analyzed in *chico* mutants (Clancy *et al*., 2002). When diets were diluted from the maximum concentration of 15% sugar and yeast, *chico*-null and wild-type genotypes robustly increased lifespan, but *chico* mutants were longest lived upon diet with 6.5% sugar and yeast, while wild-type were longest lived upon diets with 8% of these nutrients. Given these differences in the optimal diet for longevity extension and the intersection of their longevity functions in the range of more dilute diets, IIS and DR were argued to ‘act through overlapping mechanisms’ (Clancy *et al*., 2002).

Here, our aim was to further resolve whether and how IIS modulates the effect of DR upon *Drosophila* longevity. We initially approached this question by looking for diets that optimized longevity extension when dFOXO was over-expressed in fat body. We shall report that dFOXO over-expression in head fat body extended lifespan in female flies fed with a high-yeast diet, but not when fed with a restricted diet. Because *ilp2* mRNA was repressed when dFOXO extended lifespan on high-yeast diet, as previously reported (Hwangbo *et al*., 2004), we asked whether DR might slow down aging because it modulates this specific insulin-like peptide. We measured *ilp* mRNA in wild-type flies maintained on restricted and rich diets; unexpectedly, *ilp5* was repressed by DR but *ilp2* was not. To determine whether nutrient regulation of *ilp5* might be essential for DR to extend lifespan, we inhibited the diet-dependent change in its mRNA via RNAi and measured the capacity of DR to extend longevity. Dietary restriction worked equally well in cohorts with and without nutrient-responsive change in *ilp5*. Finally, we explored whether dFOXO was required for DR to extend lifespan. Dietary restriction was equally efficient in *dfoxo* null and wild-type. Overall, these data suggest that the mechanism by which DR slows down *Drosophila* aging is independent of insulin/IGF, consistent with data from *C. elegans* but contrary to prevailing perceptions for the fly.

## Results

### dFOXO over-expression and DR

At the onset, we determined the effect of dFOXO over-expression upon lifespan in female flies maintained on media that varied in dietary yeast. We expressed cDNA of wild-type *dfoxo* (UAS–*dfoxo*^+^) using the P{Switch}S_1_32 driver, which is an RU486-inducible Gal4 that is specific to head fat body (Roman *et al*., 2001). We previously reported data with this driver when RU486 was fed in yeast paste (Hwangbo *et al*., 2004). To quantitatively vary the amount of available dietary yeast, in the current work we presented the RU486 in agar–cornmeal–sugar–yeast media where all ingredient concentrations were held constant except for dietary yeast, which was set at 2%, 4%, 8% and 12% (w/v). RU486 fed to P{Switch}S_1_32 flies induces transgenes at both low- and high-yeast concentrations, based on assays with UAS–gfp as a reporter (Supplementary Fig. S1).

**Fig. 1:**
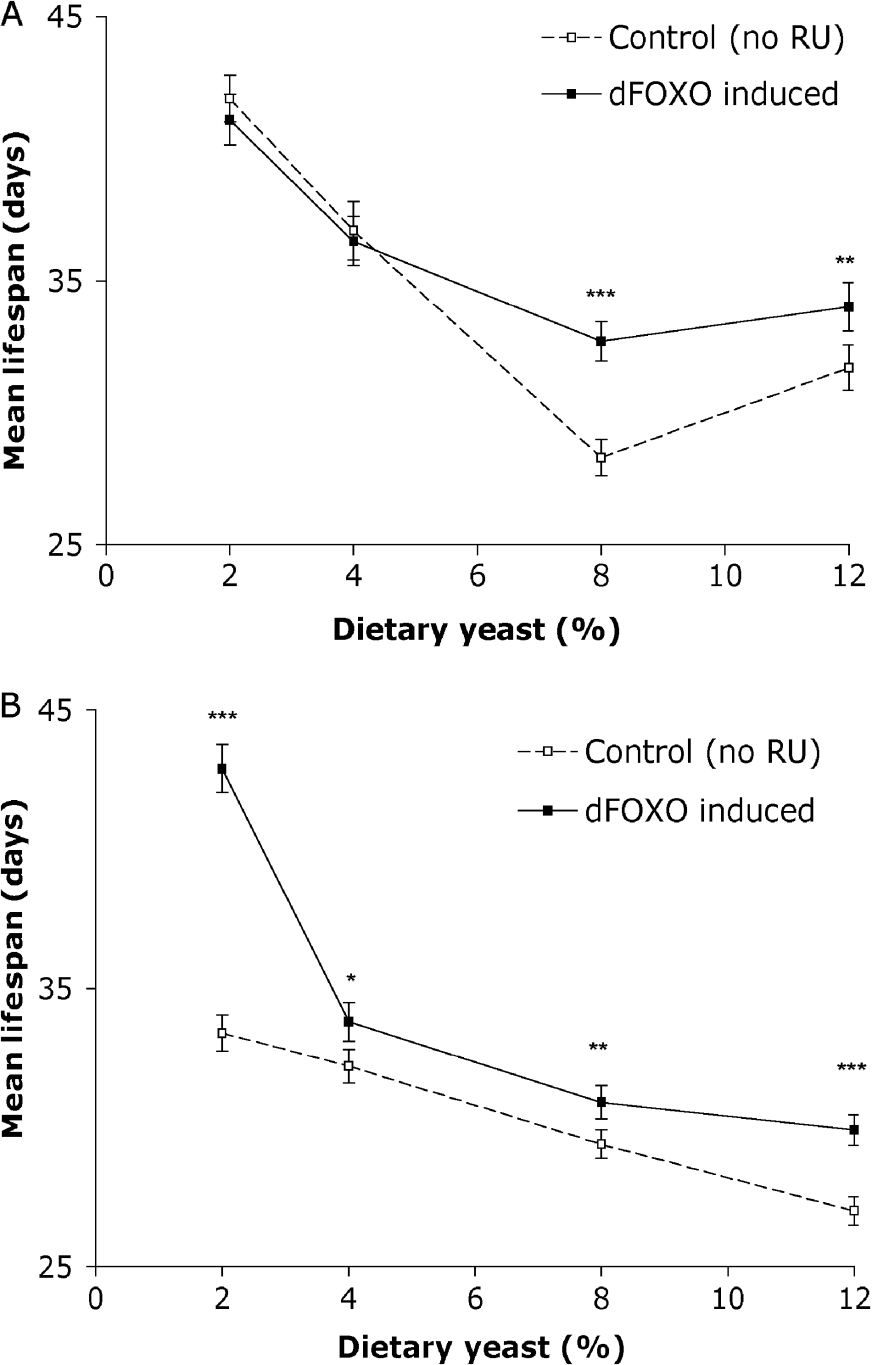
Dietary restriction in adult *Drosophila* when dFOXO is over-expressed in fat body by induction with RU486 relative to the same genotype without RU486 (vehicle only control). Mean lifespan with standard error. Asterisks indicate yeast concentrations where survival differed significantly by log-rank test (*P < 0.05; **P < 0.01; ***P < 0.0001). (A) Over-expression in the head fat body via P{Switch}S_1_32. (B) Over-expression in visceral fat body via P{Switch}S_1_106.

We measured survival for female flies maintained on a diet of each yeast concentration with or without RU486. As expected, survival of control (vehicle only) cohorts was enhanced in adults maintained upon yeast-restricted diets (Fig. 1A); mean lifespan upon 2% diet was 41.9 days while upon 12% diet it was 31.8 days (life table statistics are summarized in Table 1). Induction of UAS–*dfoxo^+^* by RU486 increased lifespan by ∼32% in female flies maintained on relatively rich diets (8% and 12%). In contrast, among flies that were long lived by virtue of DR, expression of dFOXO in head fat body via P{Switch}S_1_32 assured no additional longevity.

**Table 1:** Statistics of proportional hazard analysis for main and interaction effects of insulin peptide with diet and of FOXO with diet.

Effect	DF	χ^2^	P	Risk ratio	Lower CL	Upper CL
A. Insulin-like peptides						
Diet	1	1547.3	< 0.000	1.155	1.151	1.167
Genotype (vs. gal4/3RiA1)	2	727.72	< 0.000			
3RiA1/+				1.912	1.823	2.006
InsP3-gal4/+				0.868	0.830	0.909
Diet*genotype	2	57.29	< 0.000			
diet*geno[3RiA1/+]				1.016	1.007	1.025
diet*geno[gal4/+]				0.968	0.960	0.976
B. dFOXO						
Diet	1	906.14	< 0.000	1.226	1.210	1.242
Genotype (vs. +/+)	3	235.31	< 0.000			
*foxo*^w24^/*foxo*^21^				1.398	1.328	1.472
*foxo*^21^/+				1.116	1.056	1.181
*foxo*^w24^/+				0.773	0.734	0.874
Diet*genotype	3	36.39	< 0.000			
diet*geno[*foxo*^w24^/*foxo*^21^]				1.012	0.993	1.032
diet*geno[*foxo*^21^/+]				0.965	0.946	0.985
diet*geno[*foxo*^w24^/*+*]				0.966	0.947	0.986

The P{Switch}S_1_106 driver is expressed in thoracic and abdominal fat bodies (Roman *et al*., 2001). We previously found that expression of UAS–*dfoxo^+^* with this driver did not increase lifespan when RU486 was presented in yeast paste (Hwangbo *et al*., 2004), although a longevity benefit has been reported with RU486 delivered in sugar–yeast–agar diet (Giannakou *et al*., 2005). Here, we assess the effect of dFOXO regulated by P{Switch}S_1_106 when RU486 is presented in agar–sugar–cornmeal–yeast food media where yeast varied from 2% to 12% (Fig. 1B). At high concentrations of dietary yeast, over-expression of dFOXO increased adult survival a small amount. However, there was a 42% longevity extension with dFOXO expression in adults maintained on the most restricted diet. As a practical matter, these data show that sufficient RU486 can be consumed from restricted diets to induce FOXO-mediated longevity, providing a positive control to contrast with the absence of increased lifespan from P{Switch}S_1_32 upon restricted diets. More generally, we see that longevity extension by dFOXO depends on interactions between where the gene is expressed and the concentration of the diet.

### Messenger RNA of insulin-like peptides and DR

**Fig. 2:**
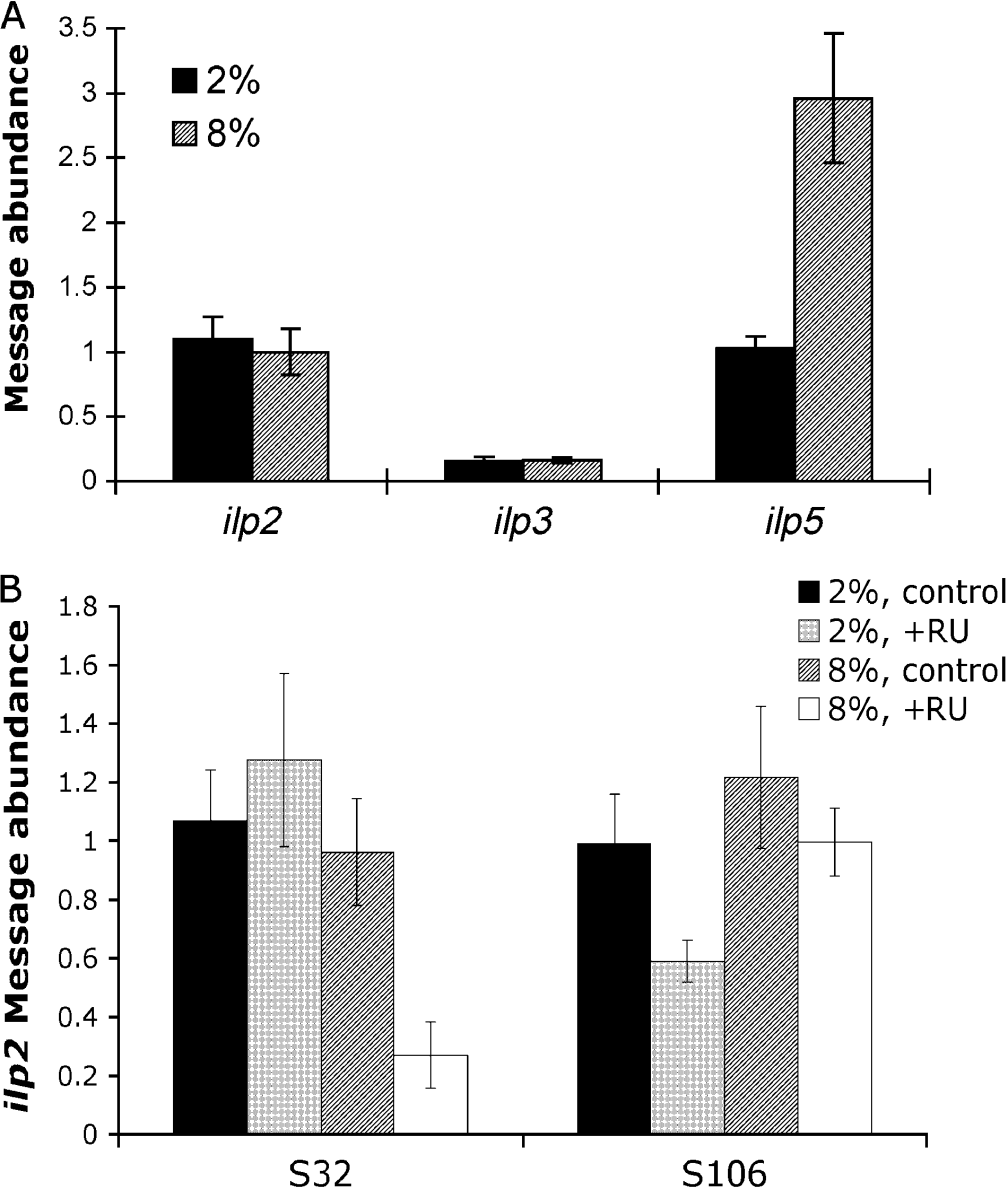
Abundance of transcripts for *insulin-like peptides* (*ilp*) isolated from adult heads. Within each replicate sample, *ilp* mRNA was normalized against mRNA of δ-tubulin. (A) mRNA of insulin-like peptide-2 (*ilp2*), *ilp3* and *ilp5* from flies maintained on 2% yeast and 8% yeast, abundance relative to *ilp2* on 2% yeast (with standard error). (B) mRNA of *ilp2* from flies on yeast concentrations of 2% and 8%, and with (+RU486) or without (control) induction of UAS–dFOXO in fat body by drivers P{Switch}S32 and P{Switch}S106.

We previously found that mRNA of *ilp2* was reduced when dFOXO was expressed in head fat body via P{Switch}S_1_32 with RU486 delivered in yeast paste (Hwangbo *et al*., 2004). Here, we consider whether reduction in *ilp2* might be responsible for the ability of DR to extend lifespan. As an initial analysis, we measured messenger RNA of insulin-like peptides from flies maintained on media with restricted (2%) and abundant (8%) dietary yeast, and with or without over-expression of dFOXO. The level of *ilp2* and *ilp3* mRNA remained constant across diets in wild-type flies, but the abundance of *ilp5* was dramatically reduced upon a restricted diet (Fig. 2A). When UAS–*dfoxo*^+^ was expressed in head fat body (via P{Switch}S_1_32), mRNA of *ilp2* was repressed in flies fed with 8% yeast, consistent with our previous observations with yeast paste, but there was no change in *ilp2* mRNA of flies upon a 2% yeast diet (Fig. 2B). When dFOXO was over-expressed from abdominal fat body (via P{Switch}S_1_106), *ilp2* was reduced upon a 2% yeast diet – the condition where dFOXO over-expression most extends lifespan – but not upon an 8% yeast diet, where there was little longevity extension (Fig. 2B).

**Fig. 3:**
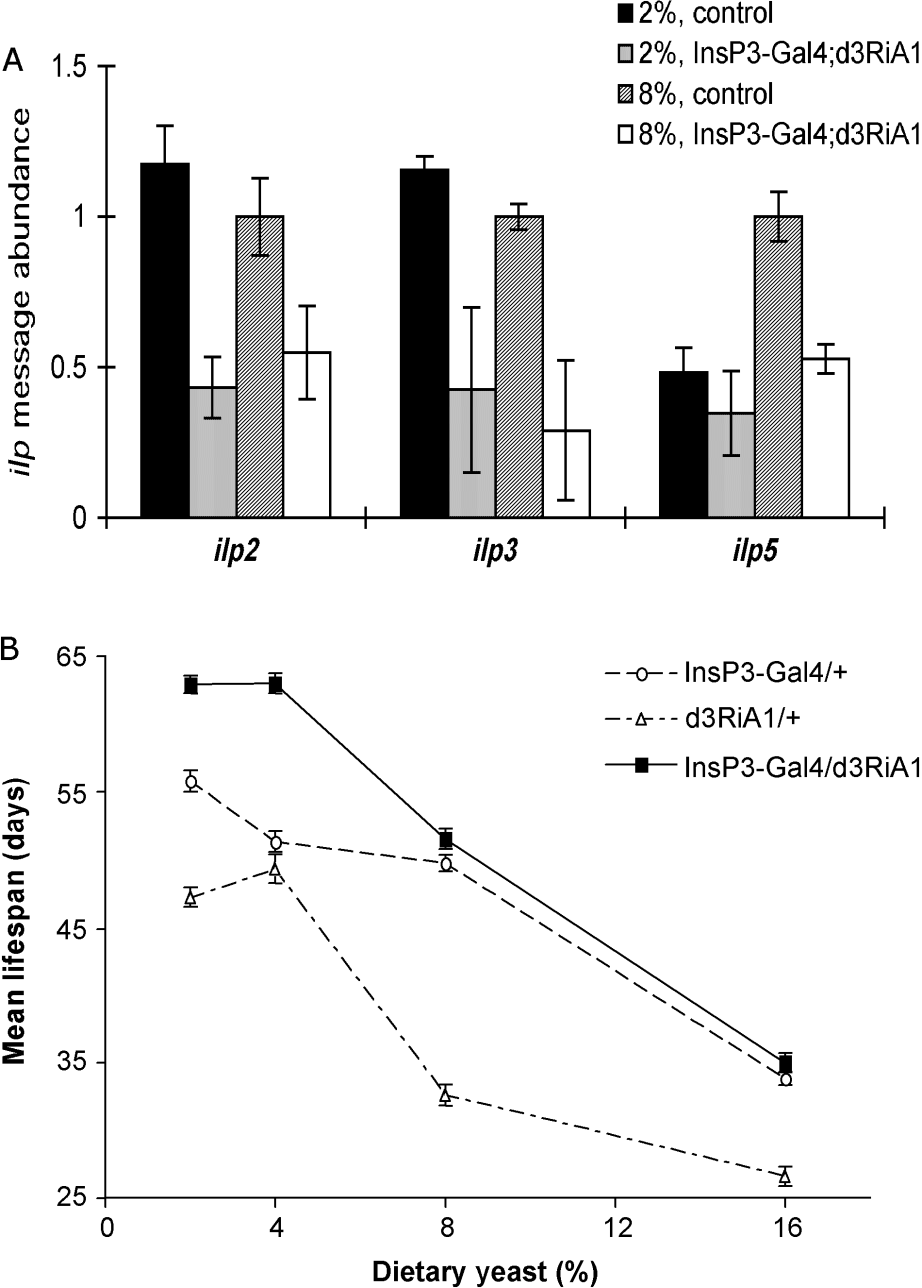
The impact of RNA interference against *insulin-like peptide*(*ilp*). (A) mRNA abundance of *ilp2*, *ilp3* and *ilp5* isolated from heads when adults were maintained on yeast concentrations of 2% and 8% (with standard error). The control genotypes were InsP3-Gal4/+ and d3RiA1/+0. The interference genotype was d3RiA1; InsP3-Gal4. Within each sample, *ilp* mRNA was normalized by the abundance of δ-tubulin, and abundance is expressed relative to the quantity in control flies upon 8% yeast. (B) Mean lifespan (standard error) as a function of dietary yeast concentration in control and interference genotypes.

To directly assess whether regulation of *ilp5* mRNA is required for DR to extend lifespan, we repressed *ilp5* with RNAi and measured survival in a range of diet concentrations. An RNAi construct (UAS–d3RiA1) designed to suppress mRNA of *ilp3* was found to also repress mRNA of *ilp2* and *ilp5* (Buch *et al*., 2007). We induced this construct in MSN from a driver with promoter sequence of *ilp3* (InsP3-Gal4) that is expressed in late third instars and adults (Buch *et al*., 2007). Induction of the RNAi construct reduced *ilp* mRNA to a consistent low level across diets (Fig. 3A). Importantly, under these conditions, *ilp5* mRNA no longer varied in response to reduction of dietary yeast, permitting us to determine whether loss of response at this specific *ilp* affected how DR extends lifespan. The response of lifespan to yeast concentration was strikingly similar among genotypes (Fig. 3B). We used survival regression with proportional hazard analysis (Parmer & Machin, 1995) to formally estimate the impact of genotype, diet and their interaction upon survival (Tatar, 2007). Diet restriction improved survival: on average, mortality increased by a factor of 1.16 per unit increase of yeast concentration (risk ratio, Table 1). Relative to the genotype with repressed *ilp* mRNA and analyzed across all diets, overall mortality was ∼2-fold higher in the 3RiA1/+control (risk ratio confidence interval: 1.82–2.01) but ∼13% less in the InsP3-Gal4/+control, despite the small advantage the *ilp*-RNAi provides in mean lifespan at some diets relative to this control (risk ratio confidence interval: 0.83–0.91) (Table 1). The differences in the effect of *ilp*-RNAi relative to the two control strains caution that background genetic effects may confound interpretations on how *ilp* reduction affects aging because overall survival should be sensitive to deleterious effects of mutation at potentially thousands of genes affecting any aspect of viability (Tatar, 1999). It is far less likely, however, for arbitrary second-site genes to affect the interaction between diet and lifespan because this is a specific rather than general phenotype. Central to the focus of our question and consistent with both controls, there was little interaction between the diet and genotype main effects; the mortality ratios for genotype-by-diet interaction did not meaningfully differ from what we would expect if loss of the *ilp5* dietary response did not affect DR (mortality ratio = 1.0) (Table 1).

### FOXO mutation and DR

**Fig. 4:**
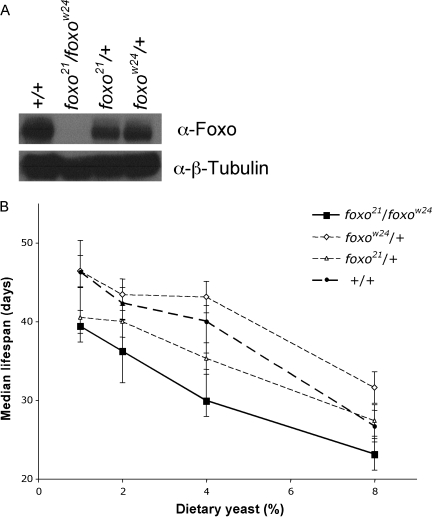
The impact of *dfoxo* mutation upon dietary restriction. (A) Western blot against dFOXO of wild-type (+/+), heterozygote (*foxo^21^*/+ and *foxo^w24^*/+), and heteroallelic null (*foxo^21^*/*foxo^w24^*) genotypes, with β-tubulin as loading control. (B) Mean lifespan (standard error) as a function of dietary yeast concentration for *dfoxo* genotypes.

To assess whether dFOXO is required for extended lifespan upon DR, we measured survival in adults with *dfoxo* wild-type and mutant genotypes maintained on media that varied in yeast concentration. We compared a loss-of-function genotype with heterozyogote and wild-type controls. The null genotype *foxo*^21^/*foxo*^w24^ produces no detectable dFOXO protein (Fig. 4A). Across four concentrations of dietary yeast, DR extends lifespan with nearly equal efficiency regardless of *dfoxo* genotype (Fig. 4B). Based on survival regression with proportional hazard analysis, DR extended lifespan with equal efficiency in flies with and without dFOXO (Table 1).

## Discussion

Dietary restriction and IIS affect *Drosophila* lifespan, and in many animals insulin signaling is the prominent endocrine response to nutrient status. From these relationships, we anticipate that mechanisms of *Drosophila* longevity extension by DR might overlap with those of IIS. Here, we evaluated this idea in several ways, but found no evidence to support the hypothesis.

Over-expression of dFOXO in adult fat bodies modulates lifespan and leads to reduction in mRNA of insulin-like ligand *ilp2* (Hwangbo *et al*., 2004). Medial secretory neurons also produce *ilp3* and *ilp5*, but neither of these change when dFOXO extends lifespan. A role for *ilp2* in longevity control has also been suggested in the analysis of aging mediated by JNK, and this effect required dFOXO (Wang *et al*., 2005). If DR modulates aging through insulin-like signals, we might expect from these observations that *ilp2* would be reduced in flies when DR extends longevity. However, we found this was not the case because *ilp5* was the only insulin-like mRNA repressed by yeast-restricted diets.

Thus, while *ilp2* appears to be associated with longevity control downstream of dFOXO and JNK, the otherwise static *ilp5* is correlated with the effect of DR upon longevity. Accordingly, we investigated whether diet-induced reduction of *ilp5* was required for DR to extend lifespan. Expression of RNAi in the MSN reduced mRNA of *ilp2*, *ilp3* and *ilp5* by approximately 50%. Importantly, this treatment eliminated the nutrient responsiveness of *ilp5*, permitting us to determine if this impaired the ability of DR to extend lifespan. We simultaneously analyzed the survival of more than 3800 adults distributed in three genotypes among four diets. Relative to fully fed adults, DR increased lifespan with equal efficiency with and without diet-responsive change in *ilp5.* We conclude that reduction of *ilp5* is not *sufficient* to extend lifespan, although we cannot rule out that diet-responsive change in *ilp5* may as yet be *necessary* for DR to modulate aging.

To investigate if a component of IIS distal to ligand synthesis contributes to DR, we evaluated whether dFOXO was required for restricted diet to extend lifespan. We compared longevity extension across four diets using 4100 flies representing confirmed, heterozygote and wild-type *dfoxo* genotypes. We found DR extends lifespan with equal efficiency with and without dFOXO.

Despite the apparent independence of insulin/IGF and DR in the control of *Drosophila* lifespan, we found that dFOXO over-expressed from fat body extends lifespan in a nutrient-dependent manner. dFOXO expressed from the head fat body only extends lifespan upon high-yeast diets, while dFOXO expressed from visceral fat body best extends lifespan upon a restricted diet. At face value, longevity extension induced by dFOXO over-expression in fully fed flies would be consistent with expectations if DR functions through IIS: dFOXO would be intrinsically induced on restricted diets, and over-expression on full diets copies this effect. This explanation, however, is not supported by our analysis with dFOXO loss-of-function genotypes. Some insight is provided by the association of reduced *ilp2* and lifespan when dFOXO is expressed in different fat bodies. Reduction of *ilp2* may increase lifespan independent of nutrition, but head and visceral fat bodies have unique conditions from which they repress brain synthesis of *ilp2*.

Our observations are consistent with current perspectives from *C. elegans* where DR efficiently extends lifespan in mutants of the insulin/IGF signaling pathway (Walker *et al*., 2005; Houthoofd *et al*., 2007; Panowski *et al*., 2007). Analysis of the interaction between DR and insulin/IGF was also studied in the context of the pituitary development mutations of mice. The Ames dwarf has deficient pituitary production of prolactin, growth hormone and thyroid-stimulating hormone, and these animals consequently have reduced circulating IGF and insulin. Because DR extends lifespan to the same extent in Ames and wild-type mice, it is thought that the mechanisms of Ames longevity assurance are independent of those induced by restricted diet (Bartke *et al*., 2001). However, mice that only lack the growth hormone response because of knockout of the growth hormone receptor locus appear to be refractory to the effect of DR upon longevity (Bonkowski *et al*., 2006). Our results are consistent with the observations from *C. elegans* and the initial conclusion from mice, but differ from the sole report of insulin/IGF and DR interactions with *Drosophila* (Clancy *et al*., 2002) where wild-type and homozygous mutants of *chico* were aged on a series of sugar–yeast diets. Lifespan of *chico* females was optimal on a diet of 8% sugar and yeast, while the longevity of wild-type flies was highest upon a diet of 6.5% sugar and yeast. In both genotypes, survival was reduced as nutrients were diluted below these optima. Importantly, the plots of longevity versus diet were parallel in the range of nutrients at concentrations greater than each genotype's optimum. These results were interpreted to represent overlap in the mechanism of insulin/IGF and DR (Clancy *et al*., 2002). However, DR occurs in the range of diets where nutrient dilution increases lifespan, not where lifespan is reduced by malnutrition. The parallelism in the range where restricted diet increases lifespan suggests that DR is equally efficient in these genotypes (Tatar, 2007), as we find for mutants of *dfoxo* and for inhibition of *ilp5* dynamics. Overall, these data suggest that the mechanisms of DR function may be independent of insulin/IGF in *Drosophila*. This argument is also consistent with recent work on diet and olfaction where sensory modulation of *Drosophila* longevity was not associated with changes in *ilp* mRNA (Libert *et al*., 2007). We are still faced, therefore, with our basic problem: how does reduced nutrient intake modulate systems that extend fly longevity?

## Experimental procedures

### Food medium and rearing conditions

Adults were collected from larvae grown on diet of cornmeal (5.2%), sugar (11.0%), autolyzed yeast (2.5%; SAF brand) and agar (0.79%) (w/v in 100 mL water) with 0.2% Tegosep (methyl 4-hydroxybenzoate, Sigma, St Louis, MO, USA). Media for adults used this standard diet except with yeast at concentrations at 1%, 2%, 4%, 8%, 12% or 16% w/v as specified in each experiment. All flies were maintained at 25 °C, 40% relative humidity and 12 h light:12 h dark. RU486 (mifepristone, Sigma) was dissolved in ethanol and added to the media at a concentration of 200 µmol; ethanol alone was added to the media of the control treatments.

### *Drosophila* stocks

Over-expression of dFOXO was studied with the genotypes *w*^1118^; UAS–*dfoxo^+^*/P{Switch}S_1_32 and +/+; UAS–*dfoxo^+^*/P{Switch}S_1_106. These were generated from crosses among the stock *w*^1118^; P{w[+mW.hs] = Switch1}S_1_32/CyO (Roman *et al*., 2001 after backcrossing to *w*^1118^; +/+; +/+, the stock+/+; P{w[+mW.hs] = Switch1}S_1_106/P{w[+mW.hs] = Switch1}S_1_106 after backcrossing to Dahomey wild-type (Giannakou *et al*., 2005), and +/+; UAS–*dfoxo*^+^/UAS–*dfoxo^+^* after backcrossing to Dahomey wild-type (Giannakou *et al*., 2005). Inhibition of *ilp* mRNA was conducted with offspring from crosses of InsP3-Gal4 (*w1118*;; *ilp3*-Gal4/*ilp3*-Gal4), d3RiA1 and *w*^1118^; +/+; +/+ (Buch *et al*., 2007). The *dilp3*-promoter construct was made by cloning an 860 bp polymerase chain reaction (PCR) fragment (primers, GCTAACTGATGATGTTTGGCCC and GACACTTGGCCAACACACACAC) into a pCaSpeRAUG-Gal4 vector. The *dilp3* RNAi construct was made by cloning a 370 bp fragment of *dilp3* (using GCATCGAGATGAGGTGTC and CTCGGCTTGGCAGC) into pCRII-TOPO-Vector (Invitrogen, Carlsbad, CA, USA), then cut with EcoRI and ligated with Sym-pUAST vector (Buch *et al*., 2007). dFOXO nulls were derived from crosses of *yw;; foxo^21^/TM6B Tb Hu e* (Junger *et al*., 2003) and *yw;; foxo^w24^/TM6B Tb Hu e* (Weber *et al*., 2005; prior to use, both stocks were backcrossed to *yw* for four generations. dFOXO heterozygote genotypes were derived from crosses of the mutant allele stocks to the *yw* stock used for the backcross.

### Analysis of *dilp* mRNA and FOXO protein

#### Western blot

Thirty 4-day-old to 5-day-old female flies were homogenized in 400 µL of loading buffer (Sambrook & Russell, 2001). Samples were boiled for 5 min and spun for 10 min; 7 µL of the extract was loaded in a 7% Tris–acetate gel (Invitrogen, EA 03552), and transferred to polyvinylidene difluoride membranes following the manufacturer's instructions. Guinea pig α-dFOXO antibody was kindly provided by Dr H. Broihier (Case Western Reserve University, Cleveland, OH, USA), and used in 1 : 5000 dilution. α−β Tubulin (hybridoma center) was used at 1 : 5000 dilution.

#### PCR

Transcript levels of *Drosophila* insulin-like peptides were measured with quantitative PCR. Live flies were frozen in liquid nitrogen and stored at –80 °C. Heads are separated using a funnel with fine mesh. Because heads can rapidly thaw and then loose RNA, all sample preparations were conducted with iced reagents and containers prior to RNAase inactivation. Total RNA from 75 heads of 7-day-old female flies was prepared using Trizol reagent (Invitrogen). RNA purity and amount were measured spectrophotometrically (NanoDrop, Thermo Scientific, Wilmington, DE, USA). DNase-treated total RNA was reverse transcribed using iScript cDNA synthesis kit (Bio-Rad, Hercules, CA, USA) according to the supplier's protocol. Real-time PCR was performed using iTaq SYBR Green Supermix with ROX (Bio-Rad) and ABI prism 7300 Sequence Detection System (Applied Biosystems, Foster City, CA, USA). mRNA of each gene was normalized relative to GAPDH2 by the method of comparative C_T_ (Livak & Schmittgen, 2001).

The primers were:

*ilp2* F ‘TGAGTATGGTGTGCGAGG’, R ‘CTCTCCACGATTCCTTGC’;
*ilp3* F ‘GAACTTTGGACCCCGTGAA’, R ‘TGAGCATCTGAACCGAACT’;
*ilp5* F ‘CAAACGAGGCACCTTGGG’, R ‘AGCTATCCAAATCCGCCA’;
*GAPDH2* F ‘GCGGTAGAATGGGGTGAGAC’, R ‘TGAAGAGCGAAAACAGTAGC’.

### Demography and survival analysis

Adult female flies were aged in 1 L demography cages. Cages were made from clear food service containers each with a ventilated lid, a gasket-covered aperture and a 25-mm-diameter plastic tube affixed to an opening along the cage side near the floor. Food vials (25 × 95 mm) were attached via the tube and changed every 2 days, at which time dead flies were removed and recorded. Female flies were collected with light CO_2_ anesthesia over a 48-h period of emergence to permit maturation and mating. Female flies were introduced at a density of 100 per demography cage. For each level of diet, at least three replicate cages were initiated per treatment.

Survival analysis was conducted with JMP statistical software with data from replicate cages combined. Kaplan–Meier methods were used to estimate mean adult lifespan with standard error. Proportional hazard analysis was used to measure how a tested genotype modified the ability of DR to increase lifespan (Tatar, 2007). Within a trial, all recorded deaths were analyzed simultaneously in a survival regression model where main effects are genotype (G) as a nominal variable and diet (D) as a continuous variable, and where the interaction parameter G × D estimates how genotype modifies the impact of diet on mortality. Survival regression with proportional hazards fits a linear model for the additive effects of these parameters, and estimates coefficients (*β_i_*) and error for each parameter *i* (Parmer & Machin, 1995). The transformation exp(*β_i_*) gives the hazard ratio, the effect upon relative mortality of one unit increment in the parameter *i*. When the hazard ratio is close to unity, there is little effect of the variable upon survival.

## Supplementary material

The following supplementary material is available for this article:

Fig. S1 UASs-gfp transgene expression is driven by P{Switch}S132 with RU486 in diets of low- and high-yeast concentration. Sets of 1-day-old to 2-day-old female flies (P{Switch}S132 > UAS-gfp) were fed media containing 2% or 8% yeast, with or without 200 µM RU486 for 5 days. Per treatment, we prepared four biological samples each with 10 heads. Samples were homogenized in 120 µL of green fluorescent protein (GFP) assay buffer (10 mM Tris-HCl pH 8.4, 100 mM NaCl, 1 mM MgCl2, 10 mM dithiothreitol), centrifuged at 10 000 r.p.m. at 4 °C and the supernatant further diluted with an additional 120 µL of GFP assay buffer. In duplicate wells, each sample was measured on a SpectraMax M5 (Molecular Devices, Sunnyvale, CA, USA) in 96-well plates and corrected for blank. RU486 induces GFP expression upon both 2% and 8% yeast diets.

This material is available as part of the online article from: http://www.blackwell-synergy.com/doi/abs/10.1111/j.1474-9726.2008.00373.x

(This link will take you to the article abstract).
