# Supplementary material for: Drosophila lifespan control by dietary restriction independent of insulin-like signaling
Source: Aging Cell. 2008 Apr 1;7(2):199–206. doi: 10.1111/j.1474-9726.2008.00373.x (PMC2340190; doi:10.1111/j.1474-9726.2008.00373.x)
Supplement: Fig. S1 — UASs-gfp transgene expression is driven by P{Switch}S132 with RU486 in diets of low- and high-yeast concentration. Sets of 1-day-old to 2-day-old female flies (P{Switch}S132 > UAS-gfp) were fed media containing 2% or 8% yeast, with or without 200 µM RU486 for 5 days. Per treatment, we prepared four biological samples each with 10 heads. Samples were homogenized in 120 µL of green fluorescent protein (GFP) assay buffer (10 mM Tris-HCl pH 8.4, 100 mM NaCl, 1 mM MgCl2, 10 mM dithiothreitol), centrifuged at 10 000 r.p.m. at 4 °C and the supernatant further diluted with an additional 120 µL of GFP assay buffer. In duplicate wells, each sample was measured on a SpectraMax M5 (Molecular Devices, Sunnyvale, CA, USA) in 96-well plates and corrected for blank. RU486 induces GFP expression upon both 2% and 8% yeast diets. [file ace0007-0199-SD1.doc]

Supplemental Material

**Drosophila life span control by dietary restriction independent of insulin-like signaling**

Kyung-Jin Min^1^ and Rochele Yamamoto^1^ (authors of equal contribution); Susanne Buch^2^, Michael Pankratz^2^, Marc Tatar^1^

Figure S1. UASs-gfp transgene expression is driven by P{Switch}S_1_32 with RU486 in diets of low and high yeast concentration.

Sets of 1 to 2 day old females (P{Switch}S_1_32 > UAS-gfp) were fed media containing 2% or 8% yeast, with or without 200 uM RU486 for five days. Per treatment we prepared four biological samples each with 10 heads. Samples were homogenized in 120 μl of GFP assay buffer (10 mM Tris.HCl pH 8.4, 100 mM NaCl, 1 mM MgCl_2_, 10 mM DTT), centrifuged at 10,000 rpm at 4°C, and the supernatant further diluted with an additional 120 μl of GFP assay buffer. In duplicate wells, each sample was measured on a Spectramax M5 in 96 well plates and corrected for blank. RU486 induces GFP expression upon both 2% and 8% yeast diets.
